# Two Cases of Liver Transplantation With a High Ionized Magnesium to Total Magnesium Ratio

**DOI:** 10.7759/cureus.23524

**Published:** 2022-03-26

**Authors:** Kunihide Okubo, Takao Kato, Yuki Shiko, Yohei Kawasaki, Ayako Inoda, Kaoru Koyama

**Affiliations:** 1 Department of Anesthesiology, Saitama Medical Center, Saitama Medical University, Kawagoe, JPN; 2 Biostatistics Section, Clinical Research Center, Chiba University Hospital, Chiba, JPN; 3 Faculty of Nursing, Japanese Red Cross College of Nursing, Tokyo, JPN; 4 Clinical Research Center, Chiba University Hospital, Chiba, JPN

**Keywords:** hypoalbuminemia, severe liver damage, critically ill patient, living liver transplantation, total magnesium, ionized magnesium, magnesium sulfate

## Abstract

Magnesium (Mg), an important cation, is involved in the activation of enzymes important for life support. The incidence of hypomagnesemia in critically ill patients admitted to the intensive care unit (ICU) is high and has been reported to be a factor in worsening prognosis. Ionized magnesium (iMg) is physiologically active, although total magnesium (tMg) is often used to evaluate the concentration of magnesium because of the limited availability of instruments that can measure iMg. However, the changes in tMg and iMg are not correlated in critically ill patients. We obtained considerable data on the simultaneous measurements of iMg and tMg in two patients with severe liver disease who underwent liver transplantation. In both patients, the iMg/tMg values were high, suggesting the influence of hypoalbuminemia associated with liver dysfunction. Mg correction using tMg as a guide may lead to overdose. Furthermore, when considering the data for each case, the correlation between iMg and tMg was very high, which suggested that the iMg/tMg ratio may be a value unique to each individual or disease. Investigating in a large-scale study the correlation between iMg levels and clinical symptoms and prognosis is necessary in the future.

## Introduction

Magnesium (Mg) is an important cation and is involved in the activation of enzymes important for life support [1]. The incidence of hypomagnesemia in critically ill patients admitted to the intensive care unit (ICU) has been reported to be 20% to 65% [2]. Ionized magnesium (iMg; standard value 0.45-0.60 mmol/L) is physiologically active, although total magnesium (tMg, standard value 0.75-1.00 mmol/L) is often used to evaluate the magnesium concentration because of the limited availability of instruments that can measure iMg [3,4]. However, the changes in tMg and iMg are not correlated in critically ill patients. The possible reasons for this finding include hypoalbuminemia, massive blood transfusion, elevated lactate levels, and the effects of concomitant medications [5-7]. To date, no study has examined multiple simultaneous measurements of iMg and tMg in the same patient over a long duration. We encountered two cases of liver transplantation in which multiple simultaneous iMg and tMg measurements were obtained during the postoperative course, and we report these data with statistical considerations.

## Case presentation

Case 1

A 50-year-old female patient with hypertension and hyperlipidemia was diagnosed with primary biliary cholangitis by her family doctor. Her preoperative Model for End-Stage Liver Disease (MELD) score which consists of serum bilirubin and creatinine levels, international normalized ratio (INR) for prothrombin time, and etiology of liver disease was 21 points [8]. She underwent a living donor liver transplant (LDLT) because she had poorly controlled ascites and hyperbilirubinemia. The operation took 18 hours. The patient was admitted to the ICU for postoperative management without extubation after surgery. During the postoperative period, catecholamines were adjusted to maintain the systolic blood pressure above 100 mmHg. Catecholamines required a maximum of 4 μg/kg/minute of dobutamine, 0.24 μg/kg/minute of noradrenaline, and 1 U/hour of vasopressin, thereby maintaining portal blood flow which was monitored daily by the surgeon using ultrasound. Moreover, 5% albumin and fresh frozen plasma were primarily used to correct ascites and drainage volume. Although his preoperative serum albumin was 2.1 g/dL, it was corrected accordingly and remained at approximately 3.0 g/dL. Magnesium was corrected, as appropriate, to maintain normal iMg levels (i.e., 0.45-0.6 mmol/L).

The patient was extubated on postoperative day 1 (POD 1). However, on POD 7, fever over 39°C and shivering were observed. Cytomegalovirus infection was suspected. On POD 8, her liver enzymes were elevated. Steroid pulse therapy was administered because acute rejection was suspected. The infection was controlled, and she was discharged from the ICU on POD 11.

**Figure 1:**
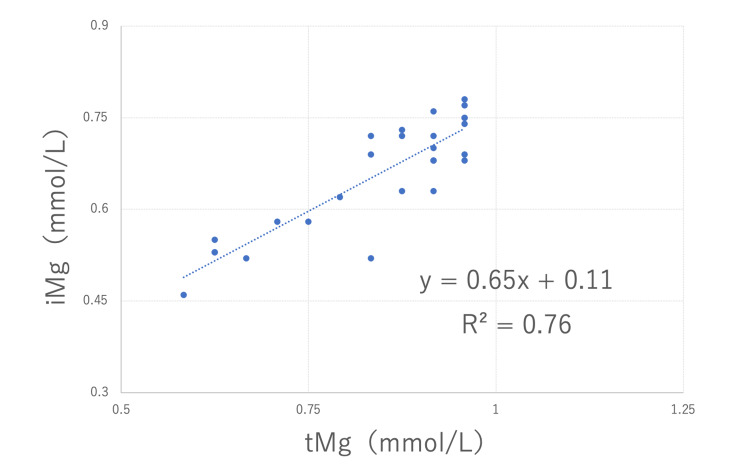
Correlation of total magnesium with ionized magnesium. In the linear regression model, the approximate equation was y = 0.65x + 0.11 and R^2^ was 0.76. tMg: total magnesium; iMg: ionized magnesium

Simultaneous measurements of iMg and tMg were taken at all 27 points during the ICU admission. The mean value of iMg/tMg was 0.76 with a standard deviation of 0.15. Only one instance occurred in which the iMg and tMg measurements were both within the standard value. The other values were relatively low for tMg. The weighted kappa coefficient was 0 (Table 1). In the linear regression model, R^2^ was 0.76 (Figure 1).

**Table 1:** Agreement between total magnesium and ionized magnesium. The tMg and iMg were classified by the lower and upper limits of their respective reference values and divided into three categories. The agreement between the two measurements in category assessment was examined with a weighted kappa coefficient and was found to be 0. tMg: total magnesium (standard value: 0.75-1.00 mmol/L); iMg: ionized magnesium (standard value: 0.45-0.60 mmol/L)

Ionized Mg category	Total Mg category			
	Low	Normal	High	Total
High	0	19	0	19
Normal	7	1	0	8
Low	0	0	0	0
Total	7	20	0	27

Case 2

A 44-year-old male patient underwent a living donor liver transplant because of alcoholic cirrhosis. The preoperative MELD score [8] was 24 points. The operation time was 10 hours. The patient was also intubated and admitted to the ICU for postoperative systemic management. He had no previous medical history. As in Case 1, the catecholamine level was adjusted based on the systolic blood pressure. We administered 5% albumin and fresh frozen plasma with reference to ascites and the drainage fluid volume. Although his preoperative serum albumin level was 2.9 g/dL, it was corrected accordingly and remained at approximately 2.7-3.5 g/dL.

He was extubated on POD 5 but underwent two reoperations for postoperative bleeding on POD 12 and POD 21. He had strong rejection. He was managed in the ICU for approximately one month. Magnesium correction was also administered in this patient with the goal of maintaining the level within the normal iMg.

**Figure 2:**
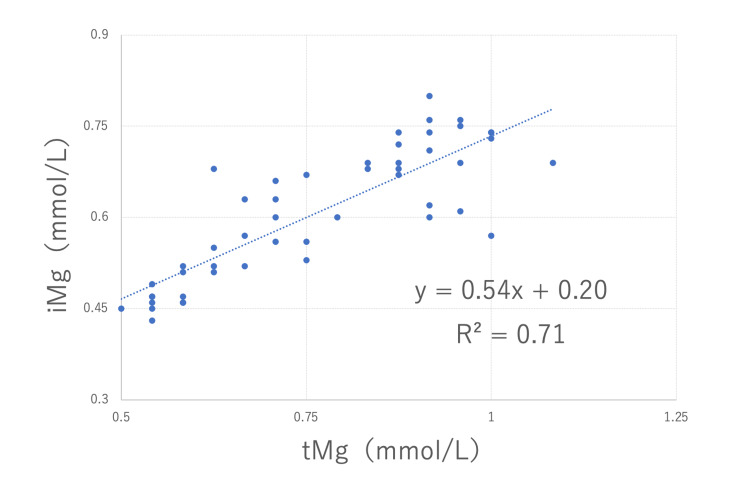
Correlation of total magnesium with ionized magnesium. In the linear regression model, the approximate equation was y = 0.54x + 0.20 and R^2 ^was 0.71. tMg: total magnesium; iMg: ionized magnesium

During the ICU admission, we measured iMg and tMg simultaneously at 56 points. The mean value of iMg/tMg was 0.81, with a standard deviation of 0.09. At 8 points, iMg and tMg were both within the standard values, and the other data had relatively low tMg. Among them, at 19 points, the tMg was low and the iMg was within the standard value. In particular, in five instances, the iMg values were high, despite low tMg values. The weighted kappa coefficient was 0.04 (Table 2). In the linear regression model, R^2^ was 0.71 (Figure 2).

**Table 2:** Agreement between total magnesium and ionized magnesium. The tMg and iMg were classified by the lower and upper limits of their respective reference values and divided into three categories. The agreement between the two measurements in category assessment was examined with a weighted kappa coefficient and was found to be 0.04. tMg: total magnesium (standard value: 0.75-1.00 mmol/L); iMg: ionized magnesium (standard value: 0.45-0.60 mmol/L)

Ionized Mg category	Total Mg category			
	Low	Normal	High	Total
High	5	22	1	28
Normal	19	8	0	27
Low	1	0	0	1
Total	25	30	1	56

## Discussion

No reports exist regarding the simultaneous measurements of iMg and tMg in the same patient. We obtained a large number of measurement data from two patients with severe liver disease who underwent liver transplantation at our hospital. We also conducted a statistical analysis. Statistical analyses were performed using the SAS statistical software package version 9.4 (SAS Institute, Cary, NC, USA).

The iMg/tMg ratio is generally 0.6-0.7 [9]. A recent randomized controlled trial reported its mean as 0.64 [10]. In critically ill patients, iMg/tMg can fluctuate in either direction and be lower and higher than the average [6]. The mean values of iMg/tMg in Case 1 and Case 2 were 0.76 and 0.81, respectively, which were much higher than previously reported values. Because this is a case series of only two cases, the cause cannot be determined. The effects of citric acid from massive blood transfusions [11], hypoalbuminemia associated with severe liver dysfunction [5], and the administration of immunosuppressive drugs [12] were considered to be possible causes. When the consistency of iMg and tMg to the standard values was verified using the weighted kappa test, the weighted kappa coefficients in Case 1 and Case 2 were 0 and 0.04, respectively, with little agreement between iMg and tMg. Similar results have been demonstrated in previous studies [6]. However, the discrepancy was more pronounced in the present two cases.

In both patients, Mg supplementation was administered at the request of the surgeon. However, the risk of Mg overdose is possible if patients are monitored for only tMg. In clinical practice, tMg is often used because of the limited availability of equipment that can measure iMg. However, if Mg is corrected using only tMg as a guide, overdose is a risk. We suggest that Mg correction using iMg measurement as an index is important, in particular, in critically ill patients with severe liver dysfunction, massive blood transfusion requirements, or the use of immunosuppressive drugs such as patients after liver transplantation.

In Case 1 and Case 2, the R^2^ was high at 0.76 and 0.71, respectively, in the linear regression model. In the present two cases, no correspondence existed between iMg and tMg when considering their consistency to the standard values. However, when considering the data for each case, the correlation was very good. Yeh et al. [6] reported a similar trend with the weighted kappa coefficient at 0.35 and R of 0.7. This finding suggested that the iMg/tMg ratio may vary between individuals or between diseases. In addition, few reports have shown a relationship between the magnesium concentration and clinical symptoms, and all of these reports used tMg data [13]. Some investigators suggest that large-scale studies are needed in the future to establish therapeutic targets and safety values for iMg concentrations, which reflect physiological activity [14].

## Conclusions

We obtained considerable data on the simultaneous measurements of iMg and tMg in two patients with severe liver disease who underwent liver transplantation. Both patients had iMg/tMg values higher than previously reported. In particular, Mg correction using tMg as a guide may lead to overdose. However, when considering the data for each case, the correlation between iMg and tMg was very high, which suggested that the iMg/tMg ratio may be a value unique to each individual or disease. Investigating in a large-scale study the correlation between iMg levels and clinical symptoms and prognosis is necessary in the future.
